# Effect of the Epley Maneuver and Brandt-Daroff Exercise on Benign Paroxysmal Positional Vertigo Involving the Posterior Semicircular Canal Cupulolithiasis: A Randomized Clinical Trial

**DOI:** 10.3389/fneur.2020.603541

**Published:** 2020-12-03

**Authors:** Seo-Young Choi, Jae Wook Cho, Jae-Hwan Choi, Eun Hye Oh, Kwang-Dong Choi

**Affiliations:** ^1^Department of Neurology, Pusan National University Hospital, Pusan National University School of Medicine and Biomedical Research Institute, Busan, South Korea; ^2^Department of Neurology, Pusan National University School of Medicine, Research Institute for Convergence of Biomedical Science and Technology, Pusan National University Yangsan Hospital, Yangsan, South Korea

**Keywords:** vertigo, nystagmus, benign paroxysmal positional vertigo, cupulolithiasis, Epley maneuver, Brandt-Daroff exercise, posterior semicircular canal

## Abstract

**Objective:** To investigate the therapeutic efficacies of the Epley maneuver and Brandt-Daroff (BD) exercise in patients with benign paroxysmal positional vertigo involving the posterior semicircular canal cupulolithiasis (PC-BPPV-cu).

**Methods:** We conducted a randomized clinical trial to evaluate the therapeutic effect of the Epley maneuver and BD exercise in patients with PC-BPPV-cu. Patients were randomly assigned to undergo the Epley maneuver (*n* = 29) or BD exercise (*n* = 33). The primary outcome was an immediate resolution of positional nystagmus within 1 h after a single treatment of each maneuver on the visit day. Secondary outcomes included the resolution of positional nystagmus at 1 week, the change of maximal slow phase velocity (mSPV) of positional nystagmus, and dizziness handicap inventory (DHI) immediately and at 1 week.

**Results:** Immediate resolution occurred in none of 29 patients in the Epley maneuver group and only 1 of 33 patients in the BD exercise group. The Epley maneuver and BD exercise had an equivalent effect at 1 week in treating PC-BPPV-cu in terms of resolving positional nystagmus (48 vs. 36%, *p* = 0.436) and the decrease of mSPV and DHI.

**Conclusion:** Neither the Epley maneuver nor BD exercise has an immediate therapeutic effect in treating PC-BPPV-cu. Clear classification of PC-BPPV should be required at the time of different pathology and different treatment response.

## Introduction

Cupulolithiasis of benign paroxysmal positional vertigo involving the posterior semicircular canal (PC-BPPV-cu) is a rare form of BPPV. Prof. Epley previously described nystagmus characteristics and his clinical experience of diagnostic posture (1). He suggested that half Hallpike maneuver can provoke persistent up and ipsitorsional nystagmus because the cupula of PC may be oriented along earth-horizontal axis, and thus the weighted cupula has maximal propensity to be deflected earthward (1). Based on his theory, Barany's society formulated the diagnostic criteria of PC-BPPV-cu on 2015 (2). PC-BPPV-cu generates upward and ipsitorsional nystagmus, but the duration of symptoms and positional nystagmus are longer (over 1 min) than experienced with canalolithiasis of PC-BPPV (PC-BPPV-ca) (1–4).

Since effective treatment of PC-BPPV-cu has not been validated, a recent clinical guideline did not recommend specific treatment options based on the subtypes of PC-BPPV (canalolithiasis or cupulolithiasis) (5, 6). Most clinics treating dizziness customarily perform diverse maneuvers for treating PC-BPPV-cu, such as the Epley maneuver, Brandt and Daroff (BD) exercise, vibratory stimulation, and head-shaking maneuver.

The BD exercise is a movement/habituation-based vestibular rehabilitation treatment and includes a sequence of rapid lateral head/trunk tilts repeated serially. This exercise could be adopted for treating cupulolithiais based on the assumption that the mechanical stimuli exerted on the cupula would help dislodge the debris from the cupula (7). However, there are no available data on the therapeutic efficacy in PC-BPPV-cu.

This study conducted a randomized clinical trial to determine the treatment efficacies of the Epley maneuver and BD exercise in patients with PC-BPPV-cu.

## Materials and Methods

### Subjects

We recruited 62 patients with a diagnosis of PC-BPPV-cu at the dizziness clinics of two university hospitals between March 2018 and October 2019. All participants met the diagnostic criteria of PC-BPPV-cu (2). Exclusion criteria included central nervous system disorders that could explain the positional vertigo and nystagmus, transition from geotropic to apogeotropic form during or after therapeutic maneuvers, multiple canals' involvement, secondary BPPV, and poor cooperation for treatments. To exclude central pathologies, all patients received neuro-otologic examinations, including spontaneous and gaze-evoked nystagmus, saccades, smooth pursuit, head impulse tests, cerebellar function tests, and assessment of balance. Patients with abnormal neurological or neuro-otological signs were referred for brain MRIs.

### Diagnostic Procedures

We performed half Dix-Hallpike maneuver and/or Dix-Hallpike maneuver to identify PC-BPPV-cu (2). The patients were also assessed with the supine head roll-test and the straight head hanging test to exclude BPPV involving horizontal or anterior canals. Nystagmus was recorded without visual fixation at a sampling rate of 120 Hz using a 3D video-oculography (SLMED, Seoul, Korea). Digitized vertical position data of the eye for maximal slow phase velocity were analyzed by the equipment software with video-oculography and verified manually.

### Study Design and Randomization

We attempted to determine therapeutic efficacies immediately and at 1 week after the Epley maneuver compared with BD exercise by a randomized clinical trial. Based on data from a previous study (8), we estimated that the proportion of patients with immediate resolution in PC-BPPV would be 80% with the Epley maneuver and 40% with the BD exercise. By adopting 0.9 power to detect a significant difference (*p* = 0.05, two-sided) and a dropout rate of 20%, we calculated that 29 patients were required for each treatment arm.

**Figure 1:**
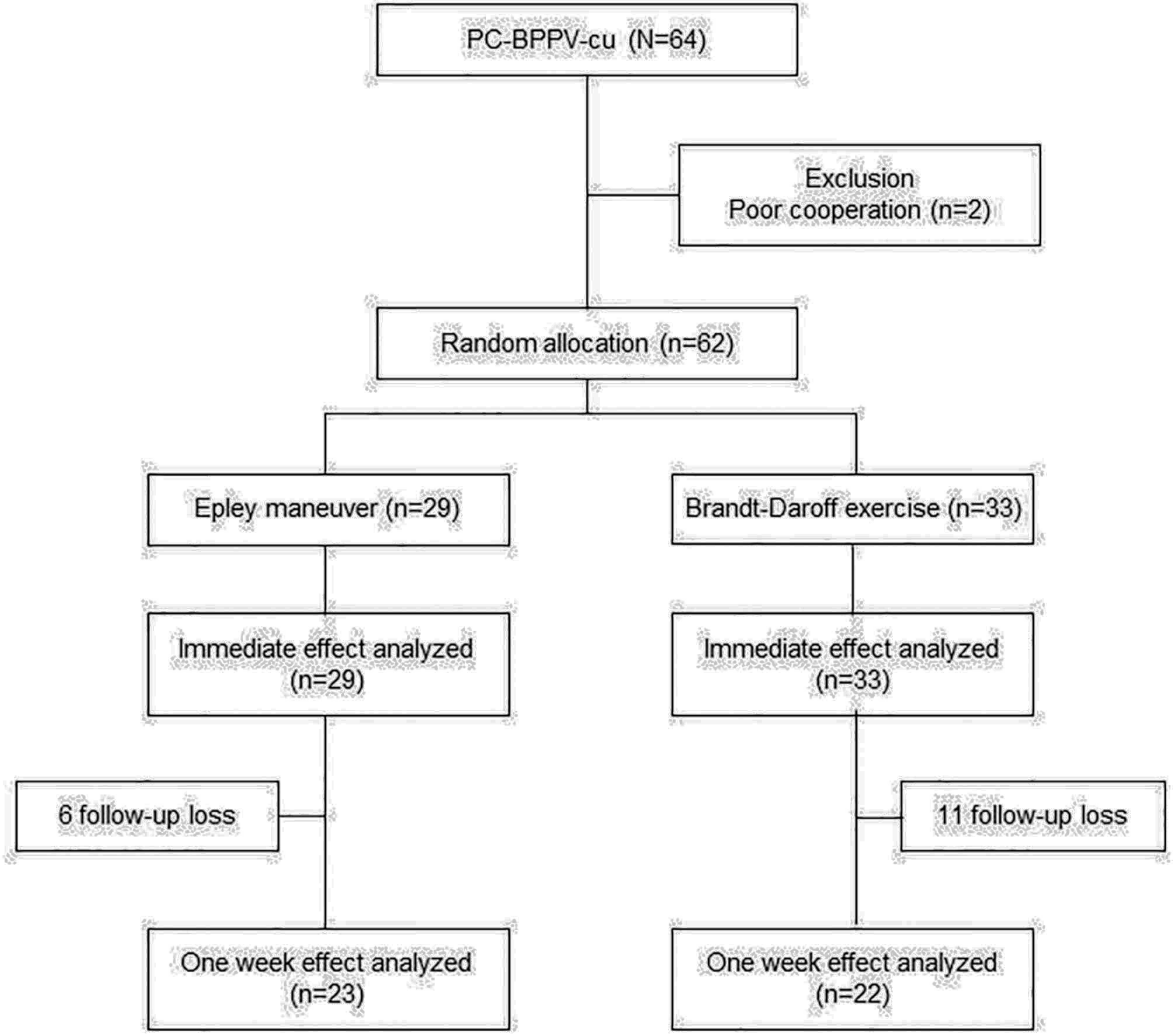
Consort diagram.

The patients with PC-BPPV-cu were randomly assigned to the Epley maneuver (*n* = 29) and DB exercise (*n* = 33) groups (Figure 1) using a web-based program. All patients completed dizziness handicap inventory (DHI) on the first visit day. Trained physiotherapists performed the assigned treatment once. A non-study physician, blinded to the maneuver applied to each patient, determined the immediate efficacy within 1 h. The patients in the BD exercise group were instructed to perform the BD exercise at home three times a day for 1 week. At the end of 1 week, all patients completed a DHI and were re-assessed for positional nystagmus.

The primary outcome was the immediate resolution of positional nystagmus after a single application of each treatment. The secondary outcomes were the resolution of positional nystagmus after 1 week, the change of maximal slow phase velocity (mSPV) of positional nystagmus, and changes in the DHI immediately after treatment and at 1 week.

### Applied Treatments

For the Epley maneuver in right PC-BPPV-cu, the head was turned 45° to the patient's right while sitting upright. Then, the patient was moved from the sitting position to the supine with the head hanging for 1 min or until the right-torsional up beating nystagmus was diminished. The head was turned 90° toward the unaffected left side twice, in a nearly face down position. The patient was then brought to the sitting up position. The patients with left PC-BPPV-cu underwent treatment in the opposite direction (9).

BD exercise was performed with a trained physiotherapist on the visit day. Patients were made to lie on their side rapidly, sit up, lie on the opposite side, and then sit up again. Each position was maintained for at least 30 s (7), and repeated serially 10 times. The patients were instructed to perform this exercise themselves at home three times daily for a week.

### Statistical Analysis

Student, paired *t*-test, or Mann-Whitney U-test was used to compare the continuous variables, and Fisher's exact-test or χ^2^-test was applied for the categorical variables. All statistical procedures were performed using SPSS statistical software (version 23.0; SPSS, Chicago, IL, USA) and *p* < 0.05 was significant.

### Standard Protocol Approvals, Registrations, and Patient Consents

The trial was registered at cris.nih.go.kr (KCT0002929). This study was performed under ethical principles consistent with the Declaration of Helsinki. The protocol and informed consent were reviewed and approved by the corresponding health authorities and ethics boards/institutional review boards for both participating study sites (1802-023-064 and 05-2018-076). Enrolled patients gave written informed consent before participation in the trial.

### Data Availability

Anonymized data will be shared by request from any qualified investigator.

## Results

### Demographic Characteristics

Of the 64 patients with PC-BPPV-cu, 62 were included for analysis on the visit day. Two individuals were excluded because they could not receive treatment because of severe vomiting (Figure 1). The mean age was 65 years (SD = 10.6, range 31–88) and 46 (74%) were women. Clinical variables did not differ between groups with Epley maneuver (*n* = 29) and DB exercise (*n* = 33) (Table 1).

**Table 1:** Comparison of clinical findings between Epley maneuver group and Brandt-Daroff exercise group.

	**Epley maneuver (*n* = 29)**	**Brandt-Daroff exercise (*n* = 33)**	***p*-value**	**Total (*n* = 62)**
Age, year (mean ± SD)	65.8 ± 8.9	64.2 ± 12.0	0.540	65.0 ± 10.6
Sex, men/women	8/21	8/25	0.780	16/46
Direction, left/right	10/11	14/19	0.606	24/38
Duration of symptoms, days (mean ± SD)	10.9 ± 23.7	7.2 ± 10.3	0.439	8.9 ±17.8

### Immediate Efficacies

**Figure 2:**
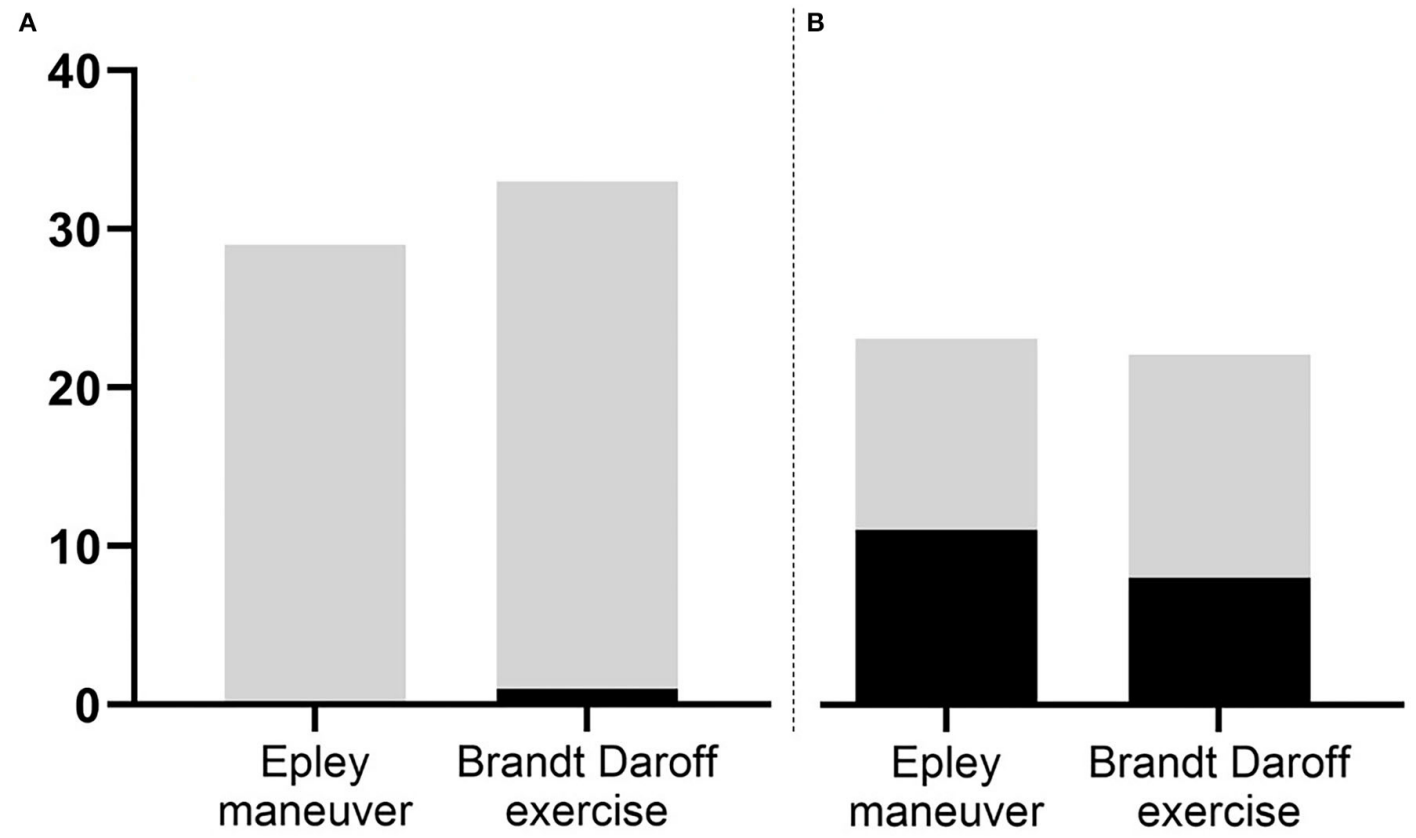
The success rate of Epley maneuver and Brandt-Daroff exercise. **(A)** On the visit day, only one patient in Brandt-Daroff exercise group shows the resolution of positional nystagmus. **(B)** At 1 week, 48% in Epley maneuver group and 36% in Brand-Daroff exercise group show the resolution of positional nystagmus without difference between two groups (*p* = 0.436, χ^2^-test). Vertical axis means the number of patients. The patients without nystagmus during Dix-Hallpike maneuver are revealed as black column, the gray column means contrary.

**Figure 3:**
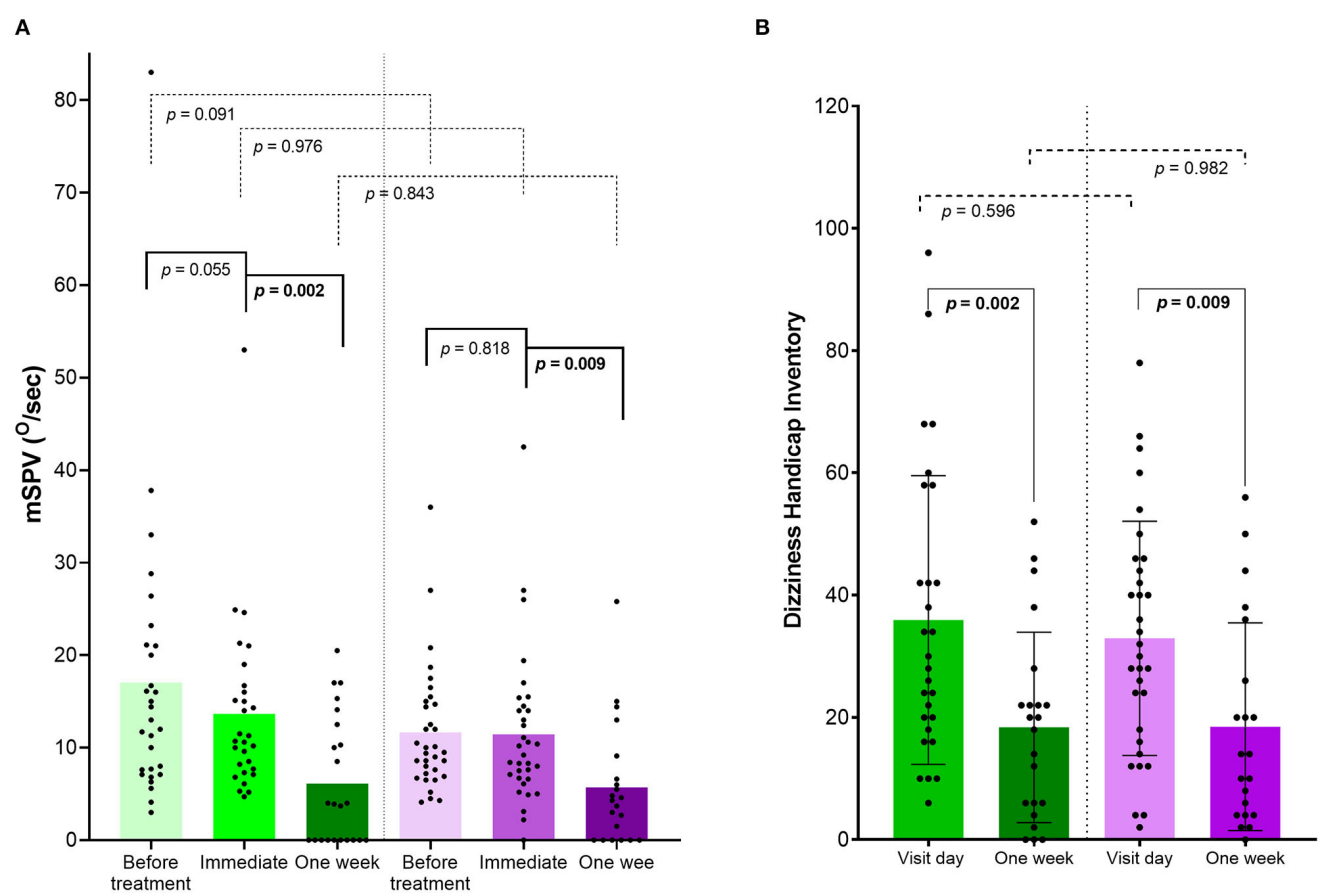
The change of maximal slow phase velocity (mSPV) of positional nystagmus **(A)** and dizziness handicap inventory (DHI) **(B)** by application of Epley maneuver and Brandt-Daroff exercise immediately and at 1 week. The mSPV does not decrease by application of both Epley maneuver (green, 17.0 ± 15.4°/s vs. 13.6 ± 9.5°/s, *p* = 0.055) and Brandt-Daroff exercise (purple, 11.6 ± 6.8°/s vs. 11.4 ± 8.2°/s, *p* = 0.818) on the visit day, but decreases at 1 week (6.1 ± 7.0°/s, *p* = 0.002 and 5.7 ± 6.6°/s, *p* = 0.009). There is no significant difference between two maneuvers before (*p* = 0.091), immediately (*p* = 0.976), as well as at 1 week (*p* = 0.843). The DHI significantly decreases at 1 week in both Epley maneuver (green, 35.9 ± 23.6 vs. 18.4 ± 15.6, *p* = 0.002) and Brandt-Daroff exercise groups (purple, 32.9 ± 19.1 vs. 18.5 ± 17.0, *p* = 0.009). There is no difference between two maneuvers on the visit day (*p* = 0.596) and at 1 week (*p* = 0.982). mSPV, maximal slow phase velocity of positional nystagmus.

After the initial maneuver, immediate resolution occurred in none of the 29 patients (0%) in the Epley maneuver group and in only 1 of 33 patients (3%) in the BD exercise group (Figure 2A). The patient showed conversion to PC-BPPV-ca. Also, there was no significant decrease in the mSPV in either of the two groups (Figure 3A).

### Response After 1 Week

After 1 week, 17 patients (17/62, 27%) were lost for follow-up, despite repeated attempts to reach them. Ultimately, the data of 45 patients were analyzed (23 with Epley maneuver and 22 with BD exercise). Clinical variables did not significantly differ between groups with Epley maneuver and DB exercise (Supplementary Table 1) and between initial and follow-up groups (data not shown). Epley maneuver and BD exercise had equivalent effect at 1 week in treating PC-BPPV-cu (48 vs. 36%, *p* = 0.436, χ^2^-test, Figure 2B). Both DHI and mSPV also significantly decreased compared to those on the first visit day, but the change did not differ between the two maneuvers (Figure 3).

## Discussion

Neither Epley maneuver nor BD exercise resulted in an improvement in PC-BPPV-cu immediately after treatment. Also, the therapeutic efficacy did not differ between the groups with Epley maneuver and BD exercise after a week, although DHI and mSPV decreased in each group.

The incidence of PC-BPPV-cu is not established, but there is a consensus that it is rare form of PC-BPPV (2). In a previous study, eight of 111 PC-BPPV (7.2%) was cupulolithiasis type (3). The authors investigated that the vertical torsional nystagmus during Dix-Hallpike test had long time constant (>40 s) while the time constant of positional nystagmus in PC-BPPC-ca was short (<20 s) (3). Also, the sum of mSPV of positional nystagmus (about 12°/s, similar to our data) was significantly lesser than that of PC-BPPV-ca (about 42°/s) (4). They explained that the force pulling cupula of moving debris in canal is greater 15 times than attached otoconia on the cupula by Pascal's principle (4).

Although Epley and Semont maneuvers are proven to be highly effective in patients with PC-BPPV-ca, research on the treatment efficacy in PC-BPPV-cu has been extremely rare. Only one observational study described the treatment efficacy in 10 patients with PC-BPPV-cu (10). They applied one each of the Semont maneuver, Epley maneuver, or hybrid maneuver (modified Semont maneuver), but none showed the resolution of positional nystagmus at 1 week, suggesting that treatment of PC-BPPV-cu would be more difficult than expected (10).

Our study is the first clinical trial to compare the therapeutic efficacy in PC-BPPV-cu of the Epley maneuver and the BD exercise. Through a randomize clinical trial, we found that neither the Epley maneuver nor the BD exercise are immediately effective for treating PC-BPPV-cu. At 1 week, there was equivalent therapeutic effect between the two maneuvers in terms of resolving positional nystagmus and decrease of mSPV and DHI. However, since our study did not adopt a control (sham) group, we could not exclude bias for the spontaneous remission. Actually, the resolution rate at 1 week in our study is like the natural course of untreated PC-BPPV, which has a spontaneous remission of 30% within 1 week (11). Our results suggest that clear classification of PC-BPPV-cu and PC-BPPV-ca should be required at the time of different pathology and different treatment response.

Future studies with a randomized, sham-controlled design are needed to validate the efficacy of various maneuvers including the Semont maneuver, head-shaking, and vibratory stimulation for the treatment of PC-BPPV-cu. Semont maneuver would be effective for PC-BPPV-cu because the maneuver use high acceleration of the head (9). Conceptually, Epley is a maneuver to redirect free otoconia in the canal, which is unlikely to be helpful in resolving cupulolithiasis. For its part, the more abrupt Semont maneuver is an unlocking maneuver for otoconia adhering to the cupola and, therefore, should be a priori more useful in cupulolithiasis. Since the addition of the pressure of the endolymph and the inertia of the heavy material in the posterior canal is theoretical base to Semont maneuver, it is not certain that Semont maneuver is more favorable to PC-BPPV-cu than–ca (12). Theoretically, the best position of PC-BPPV-cu for provocating positional nystagmus would be a Half Hallpike maneuver, because the cupula of PC may be oriented along an earth-horizontal axis during the maneuver, and thus the weighted cupula has a maximal propensity to be deflected earthward (1). Therefore, with this position maintained, application of oscillation for an extended period might settle the particles into the utricle, or the acceleration and deceleration of the head through this position may dislodge particles attached to the cupula.

Furthermore, the head-up posture during sleep for 3 months may be helpful to reduce the subjective symptoms and subjective visual vertical tilt in the intractable BPPV over 3 months, which was irrelevant to the involved semicircular canal (13). If the otolithic debris may float freely in the utricle, the head-up posture can prevent the debris to fall into the semicircular canal (14). Although it depends on the country's medical infrastructure and process, the head-up posture may be applied before the repeated maneuvers and re-visit to the hospital if the medical accessibility or the diagnosis to neurotologic specialists is not easy.

This study has several limitations. First, a relatively small number of patients was included. Second, the number of losses to follow-up was high after 1 week (27%). However, this was mitigated because clinical variables did not differ between the first and follow-up groups (Supplementary Table 1). Third, we did not execute a substitute CRM replacing the Epley or BD exercise, and a sham maneuver. Fourth, since the Brandt and Daroff exercises seek habituation, it is not logical to make an evaluation of their effectiveness and improvement of DHI immediately after the first session and even in a week. The perception of disability does not change immediately, even if the maneuver had been successful. It would have been appropriate to assess its effectiveness after at least 1 week.

In conclusion, neither the Epley maneuver nor the BD exercise improved PC-BPPV-cu immediately after treatment. A sham-controlled randomized study with a substitutional maneuver should be conducted to determine effectiveness for PC-BPPV-cu.

## Data Availability Statement

The data analyzed in this study is subject to the following licenses/restrictions: Anonymized data will be shared by request from any qualified investigator. Requests to access these datasets should be directed to Seo-Young Choi, csy035@hanmail.net.

## Ethics Statement

The protocol and informed consent were reviewed and approved by the corresponding health authorities and ethics boards/institutional review boards for all participating study sites (1802-023-064 and 05-2018-076). The patients/participants provided their written informed consent to participate in this study. Written informed consent was obtained from the individuals for the publication of any potentially identifiable images or data included in this article.

## Author Contributions

S-YC conducted the experiments, analyzed and interpreted the data, and wrote the manuscript. JC, J-HC, and EO conducted the experiments, and analyzed and interpreted the data. K-DC conducted the design and conceptualization of the study, interpretation of the data, and revised the manuscript. All authors contributed to the article and approved the submitted version.

## Conflict of Interest

The authors declare that the research was conducted in the absence of any commercial or financial relationships that could be construed as a potential conflict of interest.
