# Supplementary material for: Effect of the Epley Maneuver and Brandt-Daroff Exercise on Benign Paroxysmal Positional Vertigo Involving the Posterior Semicircular Canal Cupulolithiasis: A Randomized Clinical Trial
Source: Front Neurol. 2020 Dec 3;11:603541. doi: 10.3389/fneur.2020.603541 (PMC7793826; doi:10.3389/fneur.2020.603541)
Supplement: Supplementary file 1 [file Table_1.DOC]

**Supplemental data**

Supplemental table 1. Comparison of clinical findings between Epley maneuver group and Brandt-Daroff exercise group at 1 week.

|  | Epley maneuver  (n=23) | Brandt-Daroff exercise (n=22) | *p* value | Total (n=45) |
| --- | --- | --- | --- | --- |
| Age, year (mean ± SD) | 65.3 ± 10.6 | 64.9 ± 8.1 | 0.889 | 65.1 ± 9.4 |
| Sex, men/women | 8/15 | 6/16 | 0.586 | 14/31 |
| Direction, lef/right | 9/14 | 8/14 | 0.848 | 17/28 |
| Duration of symptoms, days (mean ± SD) | 12.8 ± 20.3 | 6.2 ± 10.1 | 0.175 | 9.6 ± 16.3 |
